# Report from the 25th Annual Western Canadian Gastrointestinal Cancer Consensus Conference on Gastric and Gastroesophageal Cancers, Winnipeg, Manitoba, 26–27 October 2023 †

**DOI:** 10.3390/curroncol31100447

**Published:** 2024-10-07

**Authors:** Ralph Wong, Brady Anderson, Bashir Bashir, Justin Bateman, Haji Chalchal, Janine Davies, Anahita Dehmoobed, Georgia Geller, Abhijit Ghose, Sharlene Gill, Vallerie Gordon, Susan Green, Pamela Hebbard, Mussawar Iqbal, Shuying Ji, Hatim Karachiwala, Biniam Kidane, Christina Kim, Ekaterina Kosyachkova, Marianne Krahn, Tharani Krishnan, Mark Kristjanson, Sangjune Lee, Richard Lee-Ying, Stephanie Lelond, Hong-Wei Liu, Daniel Meyers, Karen Mulder, James Paul, Elvira Planincic

**Affiliations:** 1 CancerCare Manitoba, Winnipeg, MB R3E 0V9, Canada; bbashir1@cancercare.mb.ca (B.B.); adehmoobed@cancercare.mb.ca (A.D.); vgordon1@cancercare.mb.ca (V.G.); sgreen3@cancercare.mb.ca (S.G.); phebbard@cancercare.mb.ca (P.H.); mkrahn@cancercare.mb.ca (M.K.); mkristjanson@cancercare.mb.ca (M.K.); slelond2@cancercare.mb.ca (S.L.); dmeyers2@cancercare.mb.ca (D.M.); elvirap969@gmail.com (E.P.); jpaul@cancercare.mb.ca (J.P.); 2 Western Manitoba Cancer Center, Brandon, MB R7A 5M8, Canada; banderson6@manitoba-physicians.ca; 3 Royal Alexandra Hospital, Edmonton, AB T5H 3V9, Canada; justin.bateman@albertaprecisionlabs.ca; 4 Allan Blair Cancer Centre, Regina, SK S4T 7T1, Canada; haji.chalchal@saskcancer.ca; 5 BC Cancer Centre, Vancouver, BC V5Z 4E6, Canada; janine.davies@bccancer.bc.ca (J.D.); sgill@bccancer.bc.ca (S.G.); tharani.krishnan@bccancer.bc.ca (T.K.); 6 BC Cancer Centre, Victoria, BC V8R 6V5, Canada; ggeller@bccancer.bc.ca; 7 Chinook Regional Hospital, Lethbridge, AB T1J 1W5, Canada; 8 Saskatchewan Cancer Agency, Regina, SK S4T 7T1, Canada; mussawar.iqbal@saskcancer.ca; 9 Shared Health Manitoba, Winnipeg, MB R2N 0E2, Canada; sji@sharedhealthmb.ca; 10 Tom Baker Cancer Centre, Calgary, AB T2N 4N2, Canada; hatim.karachiwala@ahs.ca (H.K.); richard.lee-ying@ahs.ca (R.L.-Y.); 11 Rady Faculty of Health Sciences, Max Rady College of Medicine, University of Manitoba, Winnipeg, MB R3A 1R9, Canada; bkidane@hsc.mb.ca; 12 My Gut Feeling-Stomach Cancer Foundation of Canada, Toronto, ON M5A 4T7, Canada; e.kosyachkova@mygutfeeling.ca; 13 Department of Oncology, University of Calgary, Calgary, AB T2N 4N2, Canada; sangjune.lee@ucalgary.ca; 14 Central Alberta Cancer Center, Red Deer, AB T4N 6R2, Canada; hongwei.liu@albertahealthservices.ca; 15 Cross Cancer Institute, Edmonton, AB T6G 1Z2, Canada; karen.mulder@ahs.ca

**Keywords:** gastroesophageal cancer, gastric cancer, adjuvant, biomarker-directed therapy, systemic therapies, surgical management, HER2, predictive biomarkers, survivorship

## Abstract

The 25th Annual Western Canadian Gastrointestinal Cancer Consensus Conference (WCGCCC) was held in Winnipeg, Manitoba, on 26–27 October 2023. The WCGCCC is an interactive multidisciplinary conference that was attended by healthcare professionals from across Western Canada (British Columbia, Alberta, and Manitoba) who are involved in the care of patients with gastrointestinal cancer. Surgical, medical, and radiation oncologists; pathologists; oncology nurses; pharmacists; and a family physician in oncology (FPO) participated in presentation and discussion sessions for the purpose of developing the recommendations presented here. This consensus statement addresses current issues in the management of gastroesophageal cancers.

## 1. Terms of Reference

### 1.1. Purpose

The aim of the Western Canadian Gastrointestinal Cancer Consensus Conference (WCGCCC) is to develop the consensus opinion of oncologists, pathologists, and surgeons from across Western Canada, attempting to define best care practices and to improve care and outcomes for patients with gastrointestinal cancers.

### 1.2. Participants

The WCGCCC welcomes medical oncologists, radiation oncologist, surgical oncologists, pathologists, radiologists, gastroenterologists, and allied health professionals from Western Canada who are involved in the care of patients with gastrointestinal malignancies (Table 1). Participants are provided the clinical questions to be addressed during the consensus conference in advance (Table 2).

### 1.3. Target Audience

The recommendations presented here are written for healthcare professionals involved in the care of patients with gastroesophageal cancers.

### 1.4. Basis of Recommendations

The recommendations are based on presentation and discussion of the best available evidence. Where applicable, references are cited.

## 2. Question 1

**Perioperative versus adjuvant therapies in early-stage gastroesophageal cancers—the role of chemotherapy and radiation in addition to surgery.** What is the preferred approach for locally advanced gastroesophageal cancers?

### 2.1. Recommendations

- In early-stage gastroesophageal cancers, local therapy may be considered. Cases of T3 or node-positive disease should have neoadjuvant or perioperative therapy, as per discussion in a multidisciplinary setting.
- For Siewert type I tumors [1], preoperative concurrent chemoradiation therapy is preferred in accordance with the CROSS protocol (41.4 Gy plus paclitaxel/carboplatin). Patients with residual pathologic disease should be considered for adjuvant nivolumab.
- For Siewert type II tumors [1], preoperative concurrent chemoradiation therapy can be offered in accordance with the CROSS protocol or perioperative FLOT (infusional 5-fluorouracil (5FU), leucovorin, oxaliplatin, and docetaxel). The CROSS protocol is preferred, however, due to improved tolerability, reduced duration of treatment, patient preference, and the option for adjuvant nivolumab for residual disease.
- For Siewert type III tumors [1], perioperative FLOT is preferred, as the volume for possible radiation often precludes CROSS eligibility; however, on a case-by-case basis, CROSS can be considered based on multidisciplinary team discussion.
- In patients with deficient mismatch repair (dMMR)/high microsatellite instability (MSI-H) disease, the role of adjuvant/perioperative treatment is debatable. Retrospective studies suggest that treatment is not beneficial, so upfront surgery is likely preferred; however, these evaluations were performed prior to the FLOT era and so the approach in these patients should be discussed in multidisciplinary team rounds.
- In patients who are not candidates for perioperative treatment, adjuvant therapy can be considered on a case-by-case basis.

### 2.2. Summary of Evidence—A Medical Oncology Perspective

A multidisciplinary team composed of thoracic and general surgeons, gastroenterologists, and oncologists is required to define and provide the optimal care for a patient with gastric carcinoma. Perioperative chemotherapy should be considered for curative-intent cases. Surgical resection should be with oncologic principles.

The goals of therapy are to render the patient free of disease, delay or prevent recurrence, and to improve or prolong survival. In patients that cannot be rendered disease-free, the goals are to prolong survival, to palliate symptoms, and to maximize quality of life.

Optimal care for patients with gastric adenocarcinoma requires a multidisciplinary approach involving surgery, oncology, and gastroenterology.

The goals of therapy include increasing disease and overall survival. For patients in which non-curative therapy is offered, the goals of therapy include alleviation of symptoms, increase quality of life, and prolonging survival.

#### 2.2.1. Perioperative Chemoradiotherapy—CROSS Preoperative Chemoradiation

Deliver 41.4 Gy in twenty-three fractions (1.8 Gy each) with five fractions administered per week, plus paclitaxel 50 mg/m^2^ of body surface area delivered intravenously (IV) and carboplatin area under the curve (AUC) 2 mg/mL/min IV on days 1, 8, 15, 22, and 29 [2]. This protocol improves the R0 resection rate (92% versus 69%) (*p* < 0.001) and overall survival (hazard ratio (HR) 0.657, 95% CI, 0.495–0.871, *p* = 0.003) when compared to surgery alone [2]. It prolongs median survival from 24.0 months to 49.4 months and increases the one-, two-, three-, and five-year survival rates from 70% to 82%, 50% to 67%, 44% to 58%, and 34% to 47%, respectively [2]. It offers a pathological complete response (pCR) rate of 23% [2]. Of the patients enrolled, 75% had adenocarcinoma and about 24% of patients had disease at the esophagogastric junction [2].

The Checkmate 577 trial randomized patients who received preoperative CROSS chemoradiotherapy followed by surgery with residual pathological disease (>ypT1 and/or ypN1) to receive nivolumab 240 mg every 2 weeks for 16 weeks, followed by nivolumab at a dose of 480 mg every 4 weeks, or placebo, with the maximum duration of the trial intervention period of 1 year [3]. Disease-free survival (DFS) was significantly improved in the nivolumab group compared to placebo (median 22.4 months versus 11.0 months, HR for disease recurrence or death, 0.69, 96.4% CI 0.56–0.86, *p* < 0.001) [3].

Treatment with neoadjuvant chemoradiotherapy yields higher rates of pCRs and R0 resections when compared to chemotherapy [4]. Chemoradiation is also of shorter duration, and there is no need for a central line [4]. Decisions regarding the optimal neoadjuvant therapeutic modality warrants a multidisciplinary discussion that includes incorporating planned surgery, tumor anatomy, patient wishes, and comorbidities [4].

#### 2.2.2. Perioperative Chemotherapy

In the FLOT4-AIO phase III trial, FLOT (5-fluorouracil, leucovorin, oxaliplatin, and docetaxel) was superior to either ECF (epirubicin and cisplatin,5-fluorouracil) or ECX (epirubicin, cisplatin, and capecitabine). Median overall survival was 50 months versus 35 months, HR 0.77; 0.63–0.94 *p* = 0.012. Estimated overall five-year survival was 45% vs. 36% [5]. FLOT is now considered to be the standard of care.

The MAGIC trial showed that perioperative ECF compared to surgery alone for patients ECOG ≤ 1, T2-4, N0-3, and M0 improved the five-year progression-free survival (HR 0.66; 0.53–0.81, *p* < 0.001). Overall survival was increased from 23% to 36% (HR for death 0.75, 0.60–0.93, *p* = 009) [6].

A similar benefit was noted in the FNLCC/FFCD trial with two cycles of perioperative cisplatin + fluorouracil. At a median 5.7-year follow-up, perioperative chemotherapy resulted in an improved five-year disease-free survival (34% versus 19%, HR 0.65, 95% CI, 0.48–0.89, *p* = 0.003), overall survival (38% versus 24%, HR for death: 0.69, 95% CI, 0.50–0.95, *p* = 0.02), and rate of curative resection (84% versus 73%, *p* = 0.04) [7].

In patients who have not had preoperative chemotherapy, treatment options include adjuvant chemoradiation or chemotherapy. The decision between the two approaches benefits from a multidisciplinary discussion.

According to the CRITICS trial, in patients who have received preoperative chemotherapy, there is no evidence to suggest that further intensification of postoperative treatment with chemoradiation has any benefit [5].

#### 2.2.3. Adjuvant Chemoradiation

Leucovorin followed by 5-fluororuacil combined with radiotherapy improves five-year relapse-free survival from 22% to 40% (HR 1.51, CI95% 1.25–1.83, *p* < 0.001) and five-year overall survival from 26% to 40% (HR 1.32, CI95% 1.10–1.60, *p* = 0.0046) when compared with surgery alone [6].

#### 2.2.4. Adjuvant Chemotherapy

For patients who have had an adequate lymph node dissection, particularly a D2 resection without preoperative treatment, adjuvant chemotherapy alone can be considered. It may also be considered in patients ineligible for adjuvant radiation. Treatment options include the following:

- The ARTIST regimen: capecitabine + cisplatin. The 3-year DFS was 74.2% (*p* = 0.086) in the chemotherapy arm [7].
- The CLASSIC regimen: capecitabine + oxaliplatin demonstrated a 5-year disease-free survival rate of 68% compared to 53% with surgery alone (HR 0.58, 95% CI, 0.47–0.72, *p* < 0.001) [8].

#### 2.2.5. Adjuvant Chemoradiation versus Adjuvant Chemotherapy

Consider adjuvant chemoradiation for patients with 15 or fewer lymph nodes resected or with an R1 resection (microscopically positive margins). The benefit of chemoradiation over chemotherapy has not been demonstrated after a D2 resection. A multidisciplinary discussion will help guide treatment selection. The ARTIST clinical trial randomized patients after a D2 lymph node resection to receive chemoradiation (capecitabine + cisplatin + radiation) compared to chemotherapy alone (capecitabine + cisplatin) [7]. It did not demonstrate a clear benefit of chemoradiation, with 3-year DFS 78.2% versus 74.2% *p* = 0.086 [9]. In a hypothesis-generating post hoc subgroup analysis, patients with node-positive disease appeared to have a benefit from chemoradiation (3-year DFS 77.5% vs. 72.3%, adjusted HR 0.6865; 95% CI, 0.4735 to 0.9952; *p* = 0.047) [9]. The ARTIST 2 clinical trial randomized node-positive, D2-resected gastric cancer patients to S-1, S-1 plus oxaliplatin, or radiation with S-1/oxaliplatin; the trial was closed early due to futility [10]. Preliminary data on the first 543 patients showed no significant benefit for the addition of radiation, and the S-1 arm had inferior outcomes to the oxaliplatin arm [10].

Fifty-four percent of patients in the Macdonald (Intergroup Study 0116) chemoradiation study received less than a D1 lymph node resection, with only 36% having had a D1 resection and 9.6% D2, suggesting that chemoradiation may be most beneficial in the context of an inadequate lymph node dissection [10]. CALGB 80101 randomized patients with resected gastric or EGJ tumors to adjuvant bolus 5FU and leucovorin or ECF; both arms received concurrent 5FU chemoradiation. Intensification of adjuvant chemotherapy with ECF did not significantly improve overall survival compared to adjuvant 5FU [9].

A retrospective comparison of the Dutch Gastric Cancer Group—D1D2 trial suggested significant improvements in overall survival and reduced local recurrence rates with the use of chemoradiation after an R1 resection [11]. In the FLOT4-AIO phase III trial, FLOT (5-fluorouracil, leucovorin, oxaliplatin, and docetaxel) was superior to either ECF (epirubicin and cisplatin,5-fluorouracil) or ECX (epirubicin, cisplatin, and capecitabine). Median overall survival was 50 months versus 35 months, HR 0.77; 0.63–0.94 *p* = 0.012. Estimated overall five-year survival was 45% vs. 36% [12]. FLOT is now considered to be the standard of care.

The MAGIC trial showed that perioperative ECF compared to surgery alone for patients ECOG ≤ 1, T2-4, N0-3, and M0 improved the five-year progression-free survival (HR 0.66; 0.53–0.81, *p* < 0.001). Overall survival was increased from 23% to 36% (HR for death 0.75, 0.60–0.93, *p* = 009) [13].

A similar benefit was noted in the FNLCC/FFCD trial with two cycles of perioperative cisplatin + fluorouracil. At a median 5.7-year follow-up, perioperative chemotherapy resulted in an improved five-year disease-free survival (34% versus 19%, HR 0.65, 95% CI, 0.48–0.89, *p* = 0.003), overall survival (38% versus 24%, HR for death: 0.69, 95% CI, 0.50–0.95, *p* = 0.02), and rate of curative resection (84% versus 73%, *p* = 0.04) [14].

For patients who do not receive perioperative chemotherapy, a decision for therapy should have a multidisciplinary discussion. Options include adjuvant chemoradiation and chemotherapy.

In patients who have received preoperative chemotherapy, there is no evidence to suggest that postoperative treatment with chemoradiation has any benefit. [5].

#### 2.2.6. Adjuvant Chemoradiation

The Intergroup 0116 trial showed chemoradiation with 5-flouroural/leucovorin improved five-year relapse-free survival from 22% to 40% (HR 1.51, CI95% 1.25–1.83, *p* < 0.001) and five-year overall survival from 26% to 40% (HR 1.32, CI95% 1.10–1.60, *p* = 0.0046) when compared with surgery alone [6].

#### 2.2.7. Adjuvant Chemotherapy

Adjuvant chemotherapy is an option for either patients with an adequate lymph node dissection (D2 gastrectomy) or for patients ineligible for adjuvant radiation. Treatment options include the following:

- The ARTIST regimen: capecitabine + cisplatin. The 3-year DFS was 74.2% (*p* = 0.086) in the chemotherapy arm [7].
- The CLASSIC regimen: capecitabine + oxaliplatin demonstrated a 5-year disease-free survival rate of 68% compared to 53% with surgery alone (HR 0.58, 95% CI, 0.47–0.72, *p* < 0.001) [8].

#### 2.2.8. Adjuvant Chemoradiation versus Adjuvant Chemotherapy

Adjuvant chemoradiation could be considered an option for select patients with 15 or fewer lymph nodes resected or with an R1 resection (microscopically positive margins). There is no evidence showing superiority of chemoradiation over chemotherapy after a D2 resection. These patients would benefit from a multidisciplinary discussion to guide treatment selection.

The ARTIST trial did not show a benefit of chemoradiation over chemotherapy. Postoperative patients undergoing a D2 resection were randomized to receive chemoradiation (capecitabine + cisplatin + radiation) compared to chemotherapy alone (capecitabine + cisplatin) [9]. The three-year DFS 78.2% versus 74.2% *p* = 0.086 [9]. There appeared to be a potential benefit to chemoradiation in a post hoc subgroup analysis with node-positive disease (3-year DFS 77.5% vs. 72.3%, adjusted HR 0.6865; 95% CI, 0.4735 to 0.9952; *p* = 0.047) [9]. In the successor ARTIST 2 phase III trial, node-positive, D2-resected gastric cancer patients were randomized to S-1, S-1 plus oxaliplatin, or radiation with S-1/oxaliplatin. In the first 546 enrolled patients, there was no significant benefit for the addition of radiation, and the S-1 arm had inferior outcomes to the oxaliplatin arm [10].

In the Intergroup 0116 chemoradiation study, the majority of patients (54%) received less than a D1 lymph node resection, with only 36% having had a D1 resection and 9.6% a D2 resection, suggesting that chemoradiation may be most beneficial in the context of an inadequate lymph node dissection [10]. The follow-up CALGB 80101 trial compared ECF to bolus 5-flourouracil in patients with resected gastric or EGJ tumors. Both arms received concurrent 5FU chemoradiation. No significant benefit in overall survival was demonstrated. [9].

A retrospective comparison of the Dutch Gastric Cancer Group—D1D2 trial suggested significant improvements in overall survival and reduced local recurrence rates with the use of chemoradiation after an R1 resection [11].

#### 2.2.9. Perioperative Chemotherapy versus Neoadjuvant Chemoradiotherapy

Recently, the randomized phase III ESOPEC trial was presented. It compared perioperative FLOT with the CROSS regimen in patients with resectable esophageal and GEJ adenocarcinomas [15]. With a median follow-up of 55 months, the median overall survival with the FLOT regimen was 66 months compared to 37 months with CROSS HR 0.70, 0.53–0.92, *p* = 0.012. The pCR rate was lower in the CROSS arm (10%) than expected, possibly due to the lower dose of radiotherapy that was used in the study. In addition, patients in the CROSS arm did not receive adjuvant nivolumab. The final publication of the study is eagerly awaited

Currently, there is insufficient evidence regarding dMMR status to guide treatment decisions in curative-intent gastric cancer. Patients who are identified with Lynch syndrome should be referred to genetics. There is some evidence that patients with high microsatellite instability (MSI-H) disease treated with chemotherapy have poorer outcomes [16]. Patients should be discussed in multidisciplinary rounds.

### 2.3. Summary of Evidence—A Radiation Oncology Perspective

This section will outline the management of locally advanced esophageal/gastroesophageal junction (GEJ) adenocarcinoma, which is defined as tumors that invade the muscularis propria or involve regional lymph nodes but with no distant metastases (i.e., American Joint Committee on Cancer [AJCC] stage ≥ T2 or N1 and M0).

The current National Comprehensive Cancer Network guidelines (NCCN Guidelines^®^) list preoperative chemoradiation for planned esophagectomy as the preferred management of locally advanced esophageal/GEJ adenocarcinoma [17]. However, the 2022 European Society for Medical Oncology (ESMO) guidelines list perioperative chemotherapy and trimodality therapy as equal options [18]. Prior randomized trials have shown that the predominant mode of failure is distant metastatic spread at a rate of about 30% in both the CROSS and FLOT trials [19,20].

Neo-AEGIS was a randomized control trial comparing perioperative chemotherapy versus trimodality therapy in patients with locally advanced esophageal and GEJ adenocarcinoma [21]. Patients were randomized to either perioperative therapy with epirubicin, cisplatin, and flurouracil (ECF) or FLOT chemotherapy versus trimodality therapy as per the CROSS trial, with carboplatin and paclitaxel (CP) given concurrently with 41.4 Gy in 23 fractions of radiotherapy [9]. The primary outcome was overall survival with the trial eventually changed to a non-inferiority design [9]. The hypothesis was that chemotherapy may reduce rates of distant failure and that trimodality therapy would have higher rates of operative mortality and respiratory morbidity [9]. While the results were underpowered for non-inferiority, both treatments were shown to have similar overall survival (OS) and DFS [9]. The 3-year OS was 55% with perioperative therapy versus 57% with trimodality therapy [9]. While trimodality therapy had higher rates of pCR and R0 resections, this did not translate to higher DFS or OS [9]. Despite the more intense systemic therapy, there was no statistical difference in overall rates of distant metastases [9]. However, there were significantly lower rates of liver and lung metastases in the perioperative group [9]. In both arms, about 90% of patients completed the preoperative treatment but only 41% of patients completed the adjuvant chemotherapy in the perioperative arm [9]. There was no difference in the 90-day postoperative mortality rate [9]. Quality of life was worse with trimodality therapy after treatment but then equalized at the 1- and 3-year follow-up visits [9]. Overall, the study suggests equipoise between these two treatment options [9]. However, trimodality therapy appears to have lower rates of grade 3 or higher acute toxicity during the preoperative therapy compared to perioperative FLOT [2,22]. A practical approach may be to treat Siewert I–II patients with trimodality therapy and Siewert III patients with perioperative FLOT.

In an effort to reduce the rates of distant metastases, the CALGB 80,803 (Alliance) trial included induction chemotherapy followed by PET-CT restaging to guide the selection of the chemotherapy during concurrent chemoradiotherapy [23]. Patients with esophageal adenocarcinoma were treated with folinic acid, fluorouracil, and oxaliplatin (FOLFOX) or CP [17]. If they did not respond to the induction therapy, they switched over to the other chemotherapy during concurrent chemoradiotherapy to 50.4 Gy [17]. The primary endpoint was to induce a pCR rate of greater than 5% in patients who did not respond to induction chemotherapy [17]. Two hundred and fifty-seven patients enrolled in the trial [17]. There was no difference in the rates of response to either induction chemotherapy regimen, and rates of pCR were low for nonresponders, with 18% in the FOLFOX first group and 20% in the CP first group [17]. There was no difference in OS between responders and nonresponders [17]. The FOLFOX responders had a 5-year OS of 53% [17]. These results do not show any clear benefit of induction chemotherapy [24].

CheckMate 577 was a randomized control trial in patients with locally advanced esophageal adenocarcinoma or squamous cell carcinoma [3]. Patients who underwent trimodality therapy and had an incomplete pathological response were randomized 2:1 to adjuvant nivolumab or placebo [3]. The primary endpoint was DFS. With a median follow-up of 24.4 months, 532 patients were enrolled on the immunotherapy arm and 262 on the control arm [3]. Seventy-one percent of patients had adenocarcinoma [3]. DFS was significantly improved from 11.0 months to 22.4 months, and distant recurrence was reduced from 39% to 29% with immunotherapy [3]. Grade 3 or higher adverse events was 6% in the control arm and 13% with immunotherapy [3]. While OS results are pending, CheckMate 577 is already a practice-changing trial introducing immunotherapy to the management of esophageal cancer [3].

The addition of immunotherapy to perioperative chemotherapy is being explored with two large phase III randomized controlled trials on gastric and GEJ adenocarcinoma with endpoints of pCR, event-free survival (EFS), OS, and safety. The Keynote-585 trial used pembolizumab with perioperative chemotherapy, where 20% of patients had FLOT [25]. Eight hundred and four patients were randomized with a median follow-up of 4 years [19]. While pCR was improved with immunotherapy (2.0% vs. 12.9%), EFS and OS were not significantly improved [19]. There was a trend to improved EFS, with a median of 25.3 months vs. 44.4 months [19]. Grade 3 or worse adverse events were similar at 74% versus 78% [19]. Matterhorn used durvalumab and FLOT [26]. Nine hundred and forty-four patients were randomized. pCR was improved from 7% to 19% with immunotherapy. Grade 3 or worse adverse event rate was similar at 68% vs. 69%. EFS results are pending [20]. Overall, these results do not change clinical practices.

Another approach with immunotherapy is to incorporate it with trimodality therapy. The Neo-PLANET phase II single-arm trial on patients with resectable gastric or GEJ adenocarcinoma treated patients with camrelizumab (an anti-PD-1 antibody) concurrently with capecitabine-based concurrent chemoradiotherapy of 45 Gy in 25 fractions sandwiched between oxaliplatin and capecitabine (XELOX) chemotherapy [27]. Thirty-six patients were enrolled, with 89% completing neoadjuvant therapy resulting in a pCR rate of 33.3% and a 2-year PFS of 66.9% and OS of 76.1% [21]. The OS results are in the same range as the Keynote-585 study. Grade 3 or worse adverse events were relatively high at 77.8%, with the majority being due to lymphopenia [21]. Immunotherapy concurrently with trimodality therapy is another management option to explore further. Treatment toxicities will have to be carefully balanced with any improved survival outcomes.

Esophagectomy is a life-changing surgery with a 2% chance of operative mortality. Patients are willing to give up 16% of their 5-year OS to reduce the chance of requiring esophagectomy from 100% to 35% on active surveillance [28]. The SANO trial on patients with locally advanced esophageal adenocarcinoma and squamous cell carcinoma randomized patients with a clinical complete response (cCR) to neoadjuvant chemoradiotherapy to either standard surgery or active surveillance [29]. Clinical response evaluation was based on endoscopic gastroduodenoscopy (EGD), biopsies, PET-CT, and endoscopic ultrasound (EUS) [22]. The primary endpoint was OS, with a non-inferiority design of 15% at 2 years [22]. Seventy-five percent of patients had adenocarcinoma. Thirty-four percent of all patients achieved cCR [22]. A total of 198 patients were randomized to the active surveillance arm and 111 on the standard surgery arm [22]. At a median follow-up of 38 months, non-inferiority criteria were met [22]. Among patients on active surveillance, 35% had persistent cCR and 17% developed distant metastases [22]. Operative outcomes and postoperative complications were similar between the active surveillance with salvage surgery compared to upfront surgery arms [22]. Global health-related quality of life (QOL) was significantly better in the active surveillance arm in the short term but equalized with subsequent follow-up [22]. Overall, in patients with a cCR, active surveillance results in non-inferior OS, avoids surgery in at least 35% of patients, and has better short-term QOL [22]. Delayed surgery appears to be safe [22]. While these results are noteworthy, the final manuscript is still pending. At this point, a non-operative approach in operable patients should only be pursued on a clinical trial or at least a prospective registry.

In the two-dimensional radiotherapy era, the INT 0123 (Radiation Therapy Oncology Group 94-05) phase III trial compared high-dose definitive radiotherapy of 64.8 Gy versus standard dose of 50.4 Gy and showed no significant difference in locoregional failure of OS [30]. In the modern radiotherapy era with more conformal radiation, the ARTDECO study randomized patients with inoperable esophageal carcinoma to a high-dose arm with 61.6 Gy versus a standard dose of with 50.4 Gy [31]. The primary outcome was PFS. Two hundred and sixty patients were enrolled, 61% with squamous cell carcinoma and 39% with adenocarcinoma. The 3-year PFS was not significantly different, 73% vs. 70% [24]. There was no PFS benefit for either histology [24]. Grade 4 and 5 toxicities trended higher for the high-dose arm [24]. Grade 5 toxicity was 10% vs. 5% [24]. Overall, there is no benefit of dose escalation in the definitive radiotherapy setting.

In the neoadjuvant setting, Yang et al. randomized patients with locally advanced esophageal squamous cell carcinoma to either high-dose radiation 50.4 Gy vs. standard-dose radiation 41.4 Gy [32]. Primary endpoint was 2-year PFS. One hundred and forty-seven patients enrolled [25]. The pCR rate was higher in the high-dose arm, 50% vs. 32.7%, *p* = 0.10 [25]. However, the 2-year PFS and OS were not significantly different. The rates of adverse events were not significantly different. Therefore, 41.4 Gy should be the standard preoperative radiation dose. However, given the higher pCR rates with the higher radiation dose, it may be considered if the patient is being treated with non-operative intent (Yang ESMO 2023) [25]. In addition, a meta-analysis of patients with esophageal squamous cell carcinoma and adenocarcinoma suggests that a neoadjuvant dose of 40–41.4 Gy delivered with modern radiation techniques may provide the optimal therapeutic ratio [33].

The lack of improved survival outcomes with dose escalation is likely due to the increased of long-term complications to the lungs and heart. While systemic therapy can result in cardiovascular complications, radiotherapy can have multiple effects on the cardiovascular system [34]. Radiation doses to the heart and lungs need to be reduced to improve the therapeutic ratio. In the 2D era, high doses of radiation were delivered to the heart resulting in fibrosis of the myocardial tissue [35]. Modern radiotherapy with multiple beam arrangements can reduce the high-dose coverage of the heart [36]. Clinical target volume (CTV) can also be reduced as per the proposal for contouring by Thomas et al. [37]. Improved image guidance, such as the use of the magnetic resonance linear accelerator (MR Linac), can help reduce planning target volume margins. This is achieved by breath-hold gating and real-time imaging [38]. Use of proton therapy, with the unique physics properties that eliminates dose deposition beyond the target, has been shown to reduce the risk and severity of adverse events compared to photon therapy while maintaining similar PFS [34].

## 3. Question 2

**Systemic Therapy for Advanced Gastroesophageal Cancers.** *What are the optimal first- and later-line therapies for advanced gastroesophageal cancers?*

### 3.1. Recommendations

- The recommended first-line therapy for patients with esophagogastric cancers that are *human epidermal growth factor receptor-2* (HER2)-negative and programmed death-ligand 1 (PD-L1) combined positive score (CPS) of ≥1 is a combination of 5FU and platinum chemotherapy with a *programmed death-1 (PD-1*) inhibitor.
  - There is no established benefit with the addition of a PD-1 inhibitor to chemotherapy in HER2-negative and CPS < 1.
  - In patients with esophageal squamous cell cancer or dMMR disease who are not suitable for chemotherapy, immunotherapy alone may be an option.
- First-line therapy for patients with HER2-positive gastroesophageal adenocarcinomas should be a combination 5FU and platinum chemotherapy and trastuzumab. The addition of pembrolizumab to this combination is recommended in CPS ≥ 1 disease.
- Patients whose tumor is Claudin-18 isoform 2-positive (Claudin 18.2+) should be considered for the addition of zolbetuximab to standard platinum chemotherapy.
- The optimal sequencing strategy for patients with HER2-negative, Claudin18.2+, and CPS ≥ 1 gastroesophageal adenocarcinomas is uncertain.
- The recommended first-line therapy for patients with HER2-negative, CPS-negative, and Claudin 18.2-negative esophagogastric cancers is 5FU chemotherapy.
- For HER2-negative disease, second- and later-line therapy options include paclitaxel + ramucirumab, FOLFIRI (5FU, folinic acid, and irinotecan), irinotecan, and *trifluridine/tipiracil* (TAS-102).
- In HER2-positive patients, encourage access to anti-HER2 therapies in the second- and later-lines through enrollment in clinical trials when available.

### 3.2. Summary of Evidence

Prior to the results from the four phase III first-line immune checkpoint inhibitor (ICI) studies in 2021, the standard-of-care systemic therapy for advanced gastroesophageal cancers had not changed in over ten years. The OS for HER2-negative disease had remained less than 12 months with chemotherapy, and only 40 to 50% of patients received second-line therapy [39,40]. The addition of ICIs in combination with chemotherapy improved outcomes for patients in the first-line setting; however, this also brought increasing complexity to treatment decisions. At present, choosing the optimal first-line treatment for patients in the clinic is determined mainly by HER2 and PD-L1 status and tumor histology.

There are four phase III randomized controlled trials evaluating chemotherapy in combination with ICIs in the first-line setting: Checkmate-648, Checkmate-649, Keynote-590, and Keynote-859 [41,42,43,44]. The Checkmate-648 trial was a global randomized open-label phase III study performed in patients with esophageal squamous cell carcinoma only [35]. Patients were randomized to nivolumab plus cisplatin/5FU chemotherapy, nivolumab plus ipilimumab, or chemotherapy alone [35]. The primary endpoints were OS and PFS in the PD-L1-positive population [35]. Approximately half of the included patients had tumors that were PD-L1-positive (tumor PD-L1 ≥ 1%) [35]. Seventy percent of the study population was Asian, and 60% had de novo metastatic disease [35]. At the 29-month follow-up, there was a significant OS benefit seen with chemotherapy plus ICI in PD-L1-positive and all-randomized patients versus chemotherapy alone [35]. When comparing nivolumab plus ipilimumab versus chemotherapy alone, there was again an OS benefit seen with the combination ICI arm for both PD-L1-positive and all-randomized patients [35].

The Checkmate-649 trial randomized patients with gastric, GEJ, and esophageal adenocarcinoma to nivolumab plus oxaliplatin and capecitabine (CAPOX)/FOLFOX chemotherapy, nivolumab plus ipilimumab, or chemotherapy alone [36]. In this study, only 15% of patients had a tumor PD-L1 ≥ 1% [36]. Approximately 80% of participants were non-Asian, and 70% had gastric cancer [36]. Three percent of patients in each arm were MSI-H [36]. The primary endpoint was OS and PFS in tumors with a CPS ≥ 5 [36]. At the 29-month follow-up, there was a significant OS benefit with nivolumab plus chemotherapy versus chemotherapy alone in the CPS ≥ 5 subgroup and all-randomized patients [36]. An unplanned subgroup analysis showed no benefit of adding nivolumab for the subgroups of CPS <1, <5 or <10 [36].

The Keynote-590 trial included patients with esophageal or GEJ cancers (both squamous cell and adenocarcinoma histology), and 70% had esophageal squamous cell carcinoma [37]. Patients were randomized to pembrolizumab plus cisplatin/5FU chemotherapy or chemotherapy alone [37]. Approximately 50% had tumors with a CPS ≥ 10, and 50% of patients were Asian [37]. The dual primary endpoints were OS and PFS in all esophageal squamous cell carcinoma patients and those with CPS ≥ 10 [37]. This study met its primary endpoints with a survival benefit seen for the combination arm versus chemotherapy alone [37]. There was also an OS and PFS benefit seen with the addition of pembrolizumab in all patients with CPS ≥ 10 tumors and all-randomized patients [37]. In the subgroup analysis, there was no significant survival advantage with the addition of pembrolizumab in patients with CPS < 10 tumors [37].

Lastly, the Keynote-859 trial, presented at the 2023 ESMO Plenary, included patients with gastric or GEJ adenocarcinoma [38]. Patients were randomized to pembrolizumab plus fluoropyrimidine/CAPOX chemotherapy versus chemotherapy alone [38]. Most patients received CAPOX; 78% had CPS > 1 tumors, and 25% had CPS > 10 tumors [38]. Patients were predominately non-Asian, and 75–80% had gastric cancer [38]. About 5% in each arm had MSI-H tumors [38]. In the intention-to-treat population, there was an OS benefit seen with the addition of pembrolizumab versus chemotherapy alone [38]. The benefit was greater for those with CPS > 1 and CPS > 10 tumors [38]. Again, in the subgroup analysis, those with CPS < 1 tumors did not appear to benefit from the addition of an ICI [38].

Among these four trials, there is variability with regards to the specific chemotherapy and ICI used, the tumor histology and primary tumor locations included, and the primary endpoints analyzed, which adds to the complexity when implementing the evidence into clinical practice. None of the studies examined survival in PD-L1-negative patients as a primary endpoint. From the subgroup analyses, no trial shows a benefit for chemotherapy + ICI in patients with CPS < 1 tumors. The two nivolumab studies also analyzed survival in CPS < 5 tumors and again show no benefit, particularly in adenocarcinoma patients.

Based on the evidence, chemotherapy plus ICIs have been approved in the first-line setting around the world. In Canada, the approval is currently for all patients. Using a PD-L1 cutoff will exclude some patients from accessing this treatment, which may subsequently have benefits for health service resource utilization. However, it will require a consensus regarding which PD-L1 assay to use for testing and widespread training of pathologists to ensure equitable access to testing and treatment.

Another potential first-line treatment option is targeting Claudin 18.2. This is a tight-junction protein expressed on gastric mucosa cells and retained in gastric/GEJ adenocarcinoma. Zolbetuximab is a first-in-class monoclonal antibody targeting Claudin 18.2. The SPOTLIGHT trial is a randomized placebo-controlled phase III study in patients with Claudin 18.2+ gastric/GEJ adenocarcinoma [7]. Patients were randomized to zolbetuximab plus FOLFOX versus placebo plus FOLFOX. A total of 70% of included patients were non-Asian, and most had gastric cancer. After a median follow-up of 13 months, there was a significant PFS benefit seen with the addition of zolbetuximab. There was also an OS benefit seen (a secondary endpoint of the study). In the subgroup analysis, gastric cancer patients appeared to benefit more than GEJ cancer patients. Zolbetuximab was generally well tolerated, with the most common side effects being nausea, vomiting, and neutropenia. A survival benefit for this agent was also seen in the GLOW trial, which evaluated zolbetuximab plus CAPOX versus CAPOX in a similar patient cohort (although over half of participants in this trial were Asian) [8].

To summarize the first-line options for HER2-negative patients, those with CPS > 10 and >5 tumors are most likely to benefit from chemotherapy plus ICI. CPS < 1 patients are unlikely to derive a significant benefit from ICI and should receive chemotherapy alone. Patients with CPS 1–10 and Claudin 18.2+ tumors may benefit from either an ICI or zolbetuximab in addition to chemotherapy. Patient and tumor characteristics may impact the choice of treatment between these agents; for example, potentially less benefit is seen with zolbetuximab in patients with GEJ cancers.

In gastric cancer, HER2 overexpression has been reported in 20–34% of tumors, particularly in GEJ cancer and intestinal-type gastric cancer. The current standard-of-care first-line systemic treatment for these patients is with a combination of trastuzumab plus chemotherapy as per the ToGA study [45]. The Keynote-811 study evaluated the addition of pembrolizumab to this combination and previously showed an improvement in the objective response rate (ORR) [46]. The pre-planned interim analysis presented at the 2023 ESMO Congress showed an improvement in PFS with the addition of pembrolizumab, notably in patients with CPS ≥ 1 tumors [47].

For second- and later-line systemic therapy, where possible, biomarker-directed treatments should be offered. In particular, for patients with HER2-positive tumors, anti-HER2 therapies should be sought (such as via clinical trials). The DESTINY-Gastric studies provide evidence for trastuzumab deruxtecan (T-DXd) in this setting [48,49]. DESTINY-Gastric01 was an open-label phase II study in Japan/Korea of gastric/GEJ adenocarcinoma patients after two or more prior regimens [41]. Patients were randomized 2:1 to T-DXd or physician’s choice chemotherapy [41]. There was an improvement in the ORR with T-DXd (the primary endpoint) and, importantly, a 3.5-month OS benefit in this heavily pre-treated population [41]. The DESTINY-Gastric02 trial is another phase II trial in patients who have progressed on first-line trastuzumab-containing regimens [42]. The ORR in this study was 42%, and median OS was 12 months [42]. There are important toxicity considerations with this drug, in particular interstitial lung disease [42]. However, knowledge is improving for the detection and management of this as further trials continue [42]. Other potential anti-HER2 therapies include trastuzumab plus tucatinib (via the MOUNTAINEER-2 trial) [50] or zanidatamab (via the upcoming CCTG GA.4 trial) with paclitaxel and ramucirumab [51]. The use of these biomarker-directed therapies will not change suitability for paclitaxel plus ramucirumab and TAS-102 in later lines.

## 4. Question 3

**Role of PET Scans in Surgical Management of Gastric and Gastroesophageal Cancers.** What is the role of staging PET scan in the surgical management of gastroesophageal cancers?

### 4.1. Recommendations

- Fluorodeoxyglucose (FDG)—PET imaging is recommended for staging of esophageal/GEJ cancers planned for curative-intent treatment.
- PET imaging is not routinely recommended for staging of gastric cancers.
  - PET imaging may have a role in ruling out metastatic disease that may be indeterminate on cross-sectional imaging.
  - PET utility is limited in small-volume disease (<5–10 mm), mucinous tumors, and diffuse histology.
  - PET imaging does not replace the need for diagnostic laparoscopy.
- The role of post-neoadjuvant therapy PET is not well delineated.
- PET imaging is not recommended for surveillance.

### 4.2. Summary of Evidence

The use of PET in the work-up for upper gastrointestinal cancer has been well studied and incorporated into many treatment guidelines. For gastric cancer, all Western provinces have provincial guidelines stating that PET is not for routine use and in general limited to secondary work-up of findings indeterminate for metastatic disease [52,53,54,55]. For esophageal cancer, these same provinces recommend the standard use of PET scans in patients who are candidates for radical curative treatment [45,46,47,48]. Clear evidence of metastatic disease on computed tomography (CT) will preclude the need for PET. Cancer Care Ontario guidelines for these two diseases also align with the above information [56]. For GEJ cancers, Siewert 1 and 2 lesions are treated as esophageal cancer and Siewert 3 as gastric cancer.

When PET is used, it is important to understand it limitations. The following scenarios have low fidelity with PET:

1. The lesion in question must be seen on CT;
2. The sensitivity is low for lesions under 10 mm;
3. Mucinous tumors are less likely to be PET-avid, likely due to lack of glucose transporter 1 (*GLUT1*) protein;
4. Diffuse histology is also less likely to be PET-avid.

In gastric cancer, the PLASTIC study gives important data on the sensitivity and specificity of PET and specifically compared it to diagnostic laparoscopy [57]. In this study, 394 patients had no metastatic disease on CT scans and then proceeded onto their further tests [50]. Nineteen percent of patients were found to be unresectable or metastatic on laparoscopy, while three percent of patients had metastatic disease on PET [50]. Some PET patients were also positive on laparoscopy though, so only 2% of patients had positivity on PET alone [50]. This calculated out to PET having a sensitivity of 33% and a specificity of 97% [50]. Additional testing due to incidental PET findings was performed on 83 patients [50]. Overall, these test metrics are poor.

Is there a role for PET after initial staging? Assessments of surgical resectability require high-resolution cross-sectional imaging. Modern PET scanning is a functional scan paired with a low-resolution CT for anatomic localization. These PET-CT images are insufficient for the assessment of resectability in bulky tumors and do not replace the need for IV contrast-infused CT scanning. Furthermore, reassessment for distant metastatic disease in patients who have a negative baseline PET can usually be accomplished on routine CT scanning, which is cheaper and more readily accessible.

Using PET as an early indicator of response to therapy is a novel and exciting concept. Multiple small studies show that early decrease in PET activity—measured in SUV decrease of more than 35%—is a predictor of better outcomes. Using that information to inform treatment decisions, however, is immature. Similarly, using PET to identify patients who may omit esophagectomy due to pCR is an appealing concept. A meta-analysis of studies asking this question showed poor test metrics for this task (i.e., sensitivity 60%; specificity 70%) [58].

There is no evidence to support the routine use of PET scanning in the post-treatment surveillance period.

## 5. Question 4

**Surgical Approach to Early-Stage Gastroesophageal Cancers.** When should we select a minimally invasive approach for early-stage gastroesophageal cancers?

### 5.1. Recommendations

- T1a lesions should be considered for endoscopic mucosal resection (EMR) and endoscopic submucosal dissection (ESD).
- For all other cases, a minimally invasive approach in an appropriately selected patient, in a high-volume center, and with an experienced surgeon could be considered.

### 5.2. Summary of Evidence

Randomized and nonrandomized data demonstrate that in centers of excellence with appropriate expertise, a minimally invasive approach for early-stage gastroesophageal cancers can be offered and results in similar oncologic and safety outcomes with potential improvements in perioperative outcomes [59]. The data appear more robust for minimally invasive distal gastrectomy as compared to total gastrectomy [52]. In appropriately selected cases and experienced centers with experienced surgeons, a minimally invasive total gastrectomy can be offered and results in similar oncologic and safety outcomes with potential improvements in perioperative outcomes [52,60].

## 6. Question 5—Predictive Biomarkers for the Management of Advanced Gastroesophageal Cancers. What Are the Current Predictive Biomarkers in the Management of Advanced Gastroesophageal Cancers?

### 6.1. Recommendations

- The availability of timely and standardized tumor IHC testing for HER2, dMMR, PD-L1, CPS, and Claudin 18.2+ is recommended for all patients with advanced gastroesophageal cancers being considered for first-line systemic therapy.
  - Heterogeneity of PDL1 expression, assays, and cutoffs is acknowledged.
  - Testing should be performed using clinically validated assays and interpreted by adequately trained pathologists.
- Neurotrophic tyrosine receptor kinase (NTRK) testing should be available for all patients with advanced gastroesophageal cancers.
- Testing for fibroblast growth factor receptor 2 (FGFR2b) remains an investigational option. The clinical and predictive value of Epstein–Barr virus (EBV) testing is uncertain.

### 6.2. Summary of Evidence

Currently, established predictive biomarkers in advanced stage gastroesophageal carcinomas include HER2, dMMR/microsatellite instability (MSI), and PD-L1 CPS. These biomarkers are discussed below.

HER2 is an oncoprotein encoded by the *ERBB2* gene located on chromosome 17. Overexpression or amplification of HER2 can be detected in roughly 7–38% of gastric adenocarcinomas [61]. HER2 positivity is more common in tumors with intestinal morphology (as opposed to diffuse-type) and in tumors that are moderately differentiated (compared to poorly differentiated tumors).

The ToGA trial in 2010 demonstrated efficacy using trastuzumab in combination with chemotherapy for treatment of HER2-positive gastric and gastroesophageal adenocarcinomas [45]. HER2 testing is performed by IHC to assess for protein overexpression on tumor cell membranes (a score of 3+ is considered a positive result). Reflex in situ hybridization (ISH) is performed in equivocal cases (2+ on IHC) to assess for gene amplification.

There are differences in HER2 expression and scoring between breast and gastric cancers. Testing and interpretation should be performed in accordance with guidelines published by the College of American Pathologists, American Society for Clinical Pathology, and American Society of Clinical Oncology for gastric tumors [54]. Pathologist training is advised prior to signing out cases. Molecular testing can also detect HER2 amplification (e.g., next-generation sequencing (NGS)); however, this is not currently considered first-line.

In summary, HER2 testing using a validated IHC assay (with ISH for equivocal cases) should be performed in all gastric and gastroesophageal adenocarcinomas being considered for trastuzumab therapy.

Testing for dMMR protein by IHC is recommended for all gastric and gastroesophageal adenocarcinomas. MSI testing for MSI-H tumors by PCR or NGS can also be performed but is more expensive. Tumors that are dMMR/MSI-H have high mutational burdens and are more likely to respond to immune checkpoint inhibitors. This testing can also help identify patients with Lynch syndrome. In summary, MMR testing by IHC should be considered for all gastric and gastroesophageal adenocarcinomas using a validated assay.

The PD-1 receptor inhibitors nivolumab and pembrolizumab have been approved for treatment of upper gastrointestinal tract carcinomas. Companion diagnostic tests have been developed and licensed for these targeted therapies and validated in clinical trials (22C3 IHC PharmDx for pembrolizumab and 28-8 IHC PharmDx for nivolumab).

A fit-for-purpose “3D” model for PD-L1 IHC testing is recommended, in which the appropriate diagnostic test (antibody and interpretation) is used for the specific disease and drug indication. For example, treatment of gastric adenocarcinoma with nivolumab should be based on the diagnostic test validated in the Checkmate-649 clinical trial (28-8 IHC PharmDx assay, CPS score > 5) [42]. The commercially available assays (e.g., 22C3 IHC PharmDx and 28-8 IHC PharmDx) should not be used interchangeably unless proper validation has been performed [62,63].

PD-L1 and CPS tests can be difficult to interpret, with some (but not all) studies raising concerns about interobserver agreement [64,65,66]. Pathologist training is advised prior to signing out cases. Additionally, there are concerns regarding tumor heterogeneity, both within the primary tumor and between primary and metastatic sites [67,68,69,70]. This heterogeneity may lead to false-negative results depending on the tissue tested.

In summary, PD-L1 IHC testing can be performed as needed in accordance with the 3D model, in which the clinically validated diagnostic test is used for the appropriate drug and disease indication.

Claudin 18.2 is a tight-junction protein, which is expressed in gastroesophageal adenocarcinomas and is targeted by the drug zolbetuximab. Claudin 18 IHC (VENTANA CLDN18 [43-14A] RxDx) assay has been clinically validated as a biomarker for predicting response to zolbetuximab [71]. A positive result is considered to be moderate–strong membranous staining in >75% of tumor cells. Fibroblast growth factor receptor 2 (*FGFR2*) and EBV are also being investigated as potential biomarkers [72,73].

In summary, the established predictive biomarkers currently in clinical use include HER2, dMMR, and PD-L1 CPS. Claudin 18 IHC will likely be added to this list upon Health Canada’s approval. Testing for these biomarkers is recommended for advanced gastric, gastroesophageal, and esophageal carcinomas being considered for targeted therapies. Reflex ordering of biomarkers by pathologists at the time of diagnosis should be considered where feasible to optimize turnaround time.

Testing should be performed using validated assays in accordance with published laboratory guidelines. Pathologists should be trained in biomarker interpretation prior to signing out cases. Concerns regarding tumor heterogeneity remain incompletely addressed. Interobserver disagreement among pathologists also remains an issue for PD-L1 CPS.

## Figures and Tables

**Table 1:** Attendees at the 25th Annual WCGCCC.

Name	Specialty	Organization
Brady Anderson	Medical Oncologist	Western Manitoba Cancer Center
Bashir Bashir	Radiation Oncologist	CancerCare Manitoba
Justin Bateman	Pathologist	Royal Alexandra Hospital
Haji Chalchal	Medical Oncologist	Allan Blair Cancer Centre
Janine Davies	Medical Oncologist	BC Cancer Centre
Anahita Dehmoobed	Pharmacist	CancerCare Manitoba
Georgia Geller	Medical Oncologist	BC Cancer Centre
Abhijit Ghose	Radiation Oncologist	Chinook Regional Hospital
Sharlene Gill	Medical Oncologist	BC Cancer Centre
Vallerie Gordon	Medical Oncologist	CancerCare Manitoba
Susan Green	Medical Oncologist	CancerCare Manitoba
Pamela Hebbard	Surgical Oncologist	CancerCare Manitoba
Mussawar Iqbal	Medical Oncologist	Saskatchewan Cancer Agency
Shuying Ji	Pathologist	Shared Health Manitoba
Hatim Karachiwala	Medical Oncologist	Tom Baker Cancer Centre
Biniam Kidane	Foregut/Thoracic Surgical Oncologist	University of Manitoba
Christina Kim	Medical Oncologist	CancerCare Manitoba
Ekaterina Kosyachkova	Physician Assistant	My Gut Feeling
Marianne Krahn	Medical Oncologist	CancerCare Manitoba
Tharani Krishnan	Medical Oncology Fellow	BC Cancer Centre
Mark Kristjanson	Family Physician in Oncology	CancerCare Manitoba
Sangjune Lee	Radiation Oncologist	University of Calgary
Richard Lee-Ying	Medical Oncologist	Tom Baker Cancer Centre
Stephanie Lelond	Clinical Nurse Specialist	CancerCare Manitoba
Hong-Wei Liu	Radiation Oncologist	Central Alberta Cancer Centre
Daniel Meyers	Medical Oncology Resident	CancerCare Manitoba
Karen Mulder	Medical Oncologist	Cross Cancer Institute
James Paul	Medical Oncologist	CancerCare Manitoba
Elvira Planincic	Nurse	CancerCare Manitoba
Ralph Wong	Medical Oncologist	CancerCare Manitoba

**Table 2:** Clinical questions addressing specific aspects of interest in gastrointestinal cancer are addressed as part of the 25th Annual WCGCCC.

Clinical Questions (*in order of discussion*)	
1	Perioperative vs. Adjuvant Therapies in Early-stage Gastroesophageal Cancers—Medical and Radiation Oncology Perspectives. What is the preferred approach for early-stage gastroesophageal cancers?
2	Systemic Therapy for Advanced Gastroesophageal Cancers. What are the optimal first- and later-line systemic therapies for advanced gastroesophageal cancers?
3	Role of Positron Emission Tomography (PET) Scans in Surgical Management of Gastric and Gastroesophageal Cancers. What is the role of staging PET scan in the surgical management of gastroesophageal cancers?
4	Surgical Approach to Early-Stage Gastroesophageal Cancers. When should we select a minimally invasive approach for early-stage gastroesophageal cancers?
5	Predictive Biomarkers for the Management of Advanced Gastroesophageal Cancers. What are the current predictive biomarkers in the management of advanced gastroesophageal cancers?

## Data Availability

The data presented in this study are available in this article.

## References

[1] Siewert J.R., Stein H.J. (1996). Carcinoma of the gastroesophageal junction-classification, pathology and extent of resection. Dis. Esophagus.

[2] van Hagen P., Hulshof M.C., van Lanschot J.J., Steyerberg E.W., van Berge Henegouwen M.I., Wijnhoven B.P., Richel D.J., Nieuwenhuijzen G.A., Hospers G.A., Bonenkamp J.J. (2012). Preoperative chemoradiotherapy for esophageal or junctional cancer. N. Engl. J. Med..

[3] Kelly R.J., Ajani J.A., Kuzdzal J., Zander T., Van Cutsem E., Piessen G., Mendez G., Feliciano J., Motoyama S., Lièvre A. (2021). Adjuvant Nivolumab in Resected Esophageal or Gastroesophageal Junction Cancer. N. Engl. J. Med..

[4] Chan K.K.W., Saluja R., Delos Santos K., Lien K., Shah K., Cramarossa G., Zhu X., Wong R.K.S. (2018). Neoadjuvant treatments for locally advanced, resectable esophageal cancer: A network meta-analysis. Int. J. Cancer.

[5] Cats A., Jansen E.P.M., van Grieken N.C.T., Sikorska K., Lind P., Nordsmark M., Meershoek-Klein Kranenbarg E., Boot H., Trip A.K., Swellengrebel H.A.M. (2018). Chemotherapy versus chemoradiotherapy after surgery and preoperative chemotherapy for resectable gastric cancer (CRITICS): An international, open-label, randomised phase 3 trial. Lancet Oncol..

[6] Smalley S.R., Benedetti J.K., Haller D.G., Hundahl S.A., Estes N.C., Ajani J.A., Gunderson L.L., Goldman B., Martenson J.A., Jessup J.M. (2012). Updated analysis of SWOG-directed intergroup study 0116: A phase III trial of adjuvant radiochemotherapy versus observation after curative gastric cancer resection. J. Clin. Oncol..

[7] Lee J., Lim D.H., Kim S., Park S.H., Park J.O., Park Y.S., Lim H.Y., Choi M.G., Sohn T.S., Noh J.H. (2012). Phase III trial comparing capecitabine plus cisplatin versus capecitabine plus cisplatin with concurrent capecitabine radiotherapy in completely resected gastric cancer with D2 lymph node dissection: The ARTIST trial. J. Clin. Oncol..

[8] Noh S.H., Park S.R., Yang H.K., Chung H.C., Chung I.J., Kim S.W., Kim H.H., Choi J.H., Kim H.K., Yu W. (2014). Adjuvant capecitabine plus oxaliplatin for gastric cancer after D2 gastrectomy (CLASSIC): 5-year follow-up of an open-label, randomised phase 3 trial. Lancet Oncol..

[9] Fuchs C.S., Niedzwiecki D., Mamon H.J., Tepper J.E., Ye X., Swanson R.S., Enzinger P.C., Haller D.G., Dragovich T., Alberts S.R. (2017). Adjuvant Chemoradiotherapy with Epirubicin, Cisplatin, and Fluorouracil Compared with Adjuvant Chemoradiotherapy with Fluorouracil and Leucovorin After Curative Resection of Gastric Cancer: Results from CALGB 80101 (Alliance). J. Clin. Oncol..

[10] Park S.H., Lim D.H., Sohn T.S., Lee J., Zang D.Y., Kim S.T., Kang J.H., Oh S.Y., Hwang I.G., Ji J.H. (2021). A randomized phase III trial comparing adjuvant single-agent S1, S-1 with oxaliplatin, and postoperative chemoradiation with S-1 and oxaliplatin in patients with node-positive gastric cancer after D2 resection: The ARTIST 2 trial. Ann. Oncol..

[11] Dikken J.L., Jansen E.P., Cats A., Bakker B., Hartgrink H.H., Kranenbarg E.M., Boot H., Putter H., Peeters K.C., van de Velde C.J. (2010). Impact of the extent of surgery and postoperative chemoradiotherapy on recurrence patterns in gastric cancer. J. Clin. Oncol..

[12] Al-Batran S., Homann N., Schmalenberg H., Kopp H., Haag G.M., Luley K.B., Schmiegel W.H., Folprecht G., Probst S., Prasnikar N. (2017). Perioperative chemotherapy with docetaxel, oxaliplatin, and fluorouracil/leucovorin (FLOT) versus epirubicin, cisplatin, and fluorouracil or capecitabine (ECF/ECX) for resectable gastric or gastroesophageal junction (GEJ) adenocarcinoma (FLOT4-AIO): A multicenter, randomized phase 3 trial. J. Clin. Oncol..

[13] Cunningham D., Allum W.H., Stenning S.P., Thompson J.N., Van de Velde C.J., Nicolson M., Scarffe J.H., Lofts F.J., Falk S.J., Iveson T.J. (2006). Perioperative chemotherapy versus surgery alone for resectable gastroesophageal cancer. N. Engl. J. Med..

[14] Ychou M., Boige V., Pignon J.P., Conroy T., Bouché O., Lebreton G., Ducourtieux M., Bedenne L., Fabre J.M., Saint-Aubert B. (2011). Perioperative chemotherapy compared with surgery alone for resectable gastroesophageal adenocarcinoma: An FNCLCC and FFCD multicenter phase III trial. J. Clin. Oncol..

[15] Hoeppner J., Brunner T.R., Lordick F., Schmoor C., Kulemann B., Neumann U.P., Folprecht G., Keck T., Benedix F., Schemding M. (2024). Prospective randomized multicenter phase III trial comparing perioperative chemotherapy (FLOT protocol) to neoadjuvant chemoradiation (CROSS protocol) in patients with adenocarcinoma of the esophagus (ESOPEC trial). J. Clin. Oncol..

[16] Al-Batran S., Lorenzen S., Homann N., Thuss-Patience P.C., Schenk M., Lindig U., Kretzschmar A., Heuer V., Goekkurt E., Haag G.M. (2021). Pathological regression in patients with microsatellite instability (MSI) receiving perioperative atezolizumab in combination with FLOT vs. FLOT alone for resectable esophagogastric adenocarcinoma: Results from the DANTE trial of the German Gastric Group at the AIO and SAKK. Ann. Oncol..

[17] Ajani J.A., D’Amico T.A., Bentrem D.J., Chao J., Corvera C., Das P., Denlinger C.S., Enzinger P.C., Fanta P., Farjah F. (2019). Esophageal and Esophagogastric Junction Cancers, Version 2.2019, NCCN Clinical Practice Guidelines in Oncology. J. Natl. Compr. Cancer Netw..

[18] Obermannová R., Alsina M., Cervantes A., Leong T., Lordick F., Nilsson M., van Grieken N.C.T., Vogel A., Smyth E.C., ESMO Guidelines Committee (2022). Oesophageal cancer: ESMO Clinical Practice Guideline for diagnosis, treatment and follow-up. Ann. Oncol..

[19] Eyck B.M., van Lanschot J.J.B., Hulshof M.C.C.M., van der Wilk B.J., Shapiro J., van Hagen P., van Berge Henegouwen M.I., Wijnhoven B.P.L., van Laarhoven H.W.M., Nieuwenhuijzen G.A.P. (2021). Ten-year Outcome of Neoadjuvant Chemoradiotherapy Plus Surgery for Esophageal Cancer: The Randomized Controlled CROSS Trial. J. Clin. Oncol..

[20] Glatz T., Verst R., Kuvendjiska J., Bronsert P., Becker H., Hoeppner J., Kulemann B. (2020). Pattern of Recurrence and Patient Survival after Perioperative Chemotherapy with 5-FU, Leucovorin, Oxaliplatin and Docetaxel (FLOT) for Locally Advanced Esophagogastric Adenocarcinoma in Patients Treated Outside Clinical Trials. J. Clin. Med..

[21] Reynolds J.V., Preston S.R., O’Neill B., Lowery M.A., Baeksgaard L., Crosby T., Cunningham M., Cuffe S., Griffiths G.O., Parker I. (2023). Trimodality therapy versus perioperative chemotherapy in the management of locally advanced adenocarcinoma of the oesophagus and oesophagogastric junction (Neo-AEGIS): An open-label, randomised, phase 3 trial. Lancet Gastroenterol. Hepatol..

[22] Al-Batran S.E., Homann N., Pauligk C., Goetze T.O., Meiler J., Kasper S., Kopp H.G., Mayer F., Haag G.M., Luley K. (2019). Perioperative chemotherapy with fluorouracil plus leucovorin, oxaliplatin, and docetaxel versus fluorouracil or capecitabine plus cisplatin and epirubicin for locally advanced, resectable gastric or gastro-oesophageal junction adenocarcinoma (FLOT4): A randomised, phase 2/3 trial. Lancet.

[23] Goodman K.A., Ou F.S., Hall N.C., Bekaii-Saab T., Fruth B., Twohy E., Meyers M.O., Boffa D.J., Mitchell K., Frankel W.L. (2021). Randomized Phase II Study of PET Response-Adapted Combined Modality Therapy for Esophageal Cancer: Mature Results of the CALGB 80803 (Alliance) Trial. J. Clin. Oncol..

[24] Buckstein M.H., Anker C.J., Chuong M.D., Hawkins M.A., Kharofa J., Olsen J.R. (2022). CROSSing into New Therapies for Esophageal Cancer. Int. J. Radiat. Oncol. Biol. Phys..

[25] Shitara K., Rha S.Y., Wyrwicz L.S., Oshima T., Karaseva N., Osipov M., Yasui H., Yabusaki H., Afanasyev S., Park Y.K. (2024). Neoadjuvant and adjuvant pembrolizumab plus chemotherapy in locally advanced gastric or gastro-oesophageal cancer (KEYNOTE-585): An interim analysis of the multicentre, double-blind, randomised phase 3 study. Lancet Oncol..

[26] Janjigian Y.Y., Al-Batran S., Wainberg Z.A., Van Cutsem E., Molena D., Muro K., Hyung W.J., Wyrwicz L.S., Oh D., Omori T. (2023). LBA73 Pathological complete response (pCR) to durvalumab plus 5-fluorouracil, leucovorin, oxaliplatin and docetaxel (FLOT) in resectable gastric and gastroesophageal junction cancer (GC/GEJC): Interim results of the global, phase III MATTERHORN study. Ann. Oncol..

[27] Tang Z., Wang Y., Liu D., Wang X., Xu C., Yu Y., Cui Y., Tang C., Li Q., Sun J. (2022). The Neo-PLANET phase II trial of neoadjuvant camrelizumab plus concurrent chemoradiotherapy in locally advanced adenocarcinoma of stomach or gastroesophageal junction. Nat. Commun..

[28] Noordman B.J., de Bekker-Grob E.W., Coene P.P.L.O., van der Harst E., Lagarde S.M., Shapiro J., Wijnhoven B.P.L., van Lanschot J.J.B. (2018). Patients’ preferences for treatment after neoadjuvant chemoradiotherapy for oesophageal cancer. Br. J. Surg..

[29] van der Wilk B.J., Eyck B.M., Wijnhoven B.P.L., Lagarde S.M., Rosman C., Noordman B.J., Valkema M.J., Coene P.-P., Dekker J.W., Hartgrink H. (2013). LBA75 Neoadjuvant chemoradiotherapy followed by surgery versus active surveillance for oesophageal cancer (SANO-trial): A phase-III stepped-wedge cluster randomised trial. Ann. Oncol..

[30] Minsky B.D., Pajak T.F., Ginsberg R.J., Pisansky T.M., Martenson J., Komaki R., Okawara G., Rosenthal S.A., Kelsen D.P. (2002). INT 0123 (Radiation Therapy Oncology Group 94-05) phase III trial of combined-modality therapy for esophageal cancer: High-dose versus standard-dose radiation therapy. J. Clin. Oncol..

[31] Hulshof M.C.C.M., Geijsen E.D., Rozema T., Oppedijk V., Buijsen J., Neelis K.J., Nuyttens J.J.M.E., van der Sangen M.J.C., Jeene P.M., Reinders J.G. (2021). Randomized Study on Dose Escalation in Definitive Chemoradiation for Patients with Locally Advanced Esophageal Cancer (ARTDECO Study). J. Clin. Oncol..

[32] Yang Y., Wang C., Jiang Y., Zhou X., Wang S., Su D., Qiu G., Chen Q. (2023). Different Radiation Dose of Neoadjuvant Chemoradiation for Resectable Thoracic Esophageal Squamous Carcinoma: A Randomized Phase II Clinical Trial. Int. J. Radiat. Oncol. Biol. Phys..

[33] Li Y., Liu H., Sun C., Yin X., Tong J., Zhang X., Wang X., Yuan X., Zhang Z., Lu G. (2021). Comparison of Clinical Efficacy of Neoadjuvant Chemoradiation Therapy between Lower and Higher Radiation Doses for Carcinoma of the Esophagus and Gastroesophageal Junction: A Systematic Review. Int. J. Radiat. Oncol. Biol. Phys..

[34] Herrmann J. (2020). Adverse cardiac effects of cancer therapies: Cardiotoxicity and arrhythmia. Nat. Rev. Cardiol..

[35] Takagi H., Ota H., Umezawa R., Kimura T., Kadoya N., Higuchi S., Sun W., Nakajima Y., Saito M., Komori Y. (2018). Left Ventricular T1 Mapping during Chemotherapy-Radiation Therapy: Serial Assessment of Participants with Esophageal Cancer. Radiology.

[36] Yap J.C., Malhotra H.K., Yang G.Y. (2010). Intensity modulated radiation therapy in the treatment of esophageal cancer. Thorac. Cancer.

[37] Thomas M., Mortensen H.R., Hoffmann L., Møller D.S., Troost E.G.C., Muijs C.T., Berbee M., Bütof R., Nicholas O., Radhakrishna G. (2021). Proposal for the delineation of neoadjuvant target volumes in oesophageal cancer. Radiother. Oncol..

[38] Lee S.L., Mahler P., Olson S., Witt J.S., Musunuru H.B., Rajamanickam V., Bassetti M.F., Yadav P. (2021). Reduction of cardiac dose using respiratory-gated MR-linac plans for gastro-esophageal junction cancer. Med. Dosim..

[39] Smyth E.C., Nilsson M., Grabsch H.I., van Grieken N.C., Lordick F. (2020). Gastric cancer. Lancet.

[40] Wagner A.D., Syn N.L., Moehler M., Grothe W., Yong W.P., Tai B.C., Ho J., Unverzagt S. (2017). Chemotherapy for advanced gastric cancer. Cochrane Database Syst. Rev..

[41] Doki Y., Ajani J.A., Kato K., Xu J., Wyrwicz L., Motoyama S., Ogata T., Kawakami H., Hsu C.H., Adenis A. (2022). Nivolumab Combination Therapy in Advanced Esophageal Squamous-Cell Carcinoma. N. Engl. J. Med..

[42] Janjigian Y.Y., Shitara K., Moehler M., Garrido M., Salman P., Shen L., Wyrwicz L., Yamaguchi K., Skoczylas T., Campos Bragagnoli A. (2021). First-line nivolumab plus chemotherapy versus chemotherapy alone for advanced gastric, gastro-oesophageal junction, and oesophageal adenocarcinoma (CheckMate 649): A randomised, open-label, phase 3 trial. Lancet.

[43] Sun J.M., Shen L., Shah M.A., Enzinger P., Adenis A., Doi T., Kojima T., Metges J.P., Li Z., Kim S.B. (2021). Pembrolizumab plus chemotherapy versus chemotherapy alone for first-line treatment of advanced oesophageal cancer (KEYNOTE-590): A randomised, placebo-controlled, phase 3 study. Lancet.

[44] Rha S.Y., Wyrwicz L.S., Weber P.E.Y., Bai Y., Ryu M.H., Lee J., Rivera F., Alves G.V., Garrido M., Shiu K.-K. (2023). Pembrolizumab plus chemotherapy as first-line therapy for advanced HER2-negative gastric or gastroesophageal junction cancer: Phase III KEYNOTE-859 study. Ann. Oncol..

[45] Bang Y.J., Van Cutsem E., Feyereislova A., Chung H.C., Shen L., Sawaki A., Lordick F., Ohtsu A., Omuro Y., Satoh T. (2010). Trastuzumab in combination with chemotherapy versus chemotherapy alone for treatment of HER2-positive advanced gastric or gastro-oesophageal junction cancer (ToGA): A phase 3, open-label, randomised controlled trial. Lancet.

[46] Janjigian Y.Y., Kawazoe A., Yañez P., Li N., Lonardi S., Kolesnik O., Barajas O., Bai Y., Shen L., Tang Y. (2021). The KEYNOTE-811 trial of dual PD-1 and HER2 blockade in HER2-positive gastric cancer. Nature.

[47] Janjigian Y.Y., Kawazoe A., Bai Y., Xu J., Lonardi S., Metges J.P., Yanez P., Wyrwicz L.S., Shen L., Ostapenko Y. (2023). Pembrolizumab plus trastuzumab and chemotherapy for HER2-positive gastric or gastro-oesophageal junction adenocarcinoma: Interim analyses from the phase 3 KEYNOTE-811 randomised placebo-controlled trial. Lancet.

[48] Shitara K., Bang Y.J., Iwasa S., Sugimoto N., Ryu M.H., Sakai D., Chung H.C., Kawakami H., Yabusaki H., Lee J. (2020). DESTINY-Gastric01 Investigators. Trastuzumab Deruxtecan in Previously Treated HER2-Positive Gastric Cancer. N. Engl. J. Med..

[49] Van Cutsem E., di Bartolomeo M., Smyth E., Chau I., Park H., Siena S., Lonardi S., Wainberg Z.A., Ajani J., Chao J. (2023). Trastuzumab deruxtecan in patients in the USA and Europe with HER2-positive advanced gastric or gastroesophageal junction cancer with disease progression on or after a trastuzumab-containing regimen (DESTINY-Gastric02): Primary and updated analyses from a single-arm, phase 2 study. Lancet Oncol..

[50] Tucatinib, Trastuzumab, Ramucirumab, and Paclitaxel Versus Paclitaxel and Ramucirumab in Previously Treated HER2+ Gastroesophageal Cancer (MOUNTAINEER-02). https://clinicaltrials.gov/study/NCT04499924#study-record-dates.

[51] Paclitaxel and Ramucirumab +/- Zanidatamab in HER2 Postive Advanced Gastroesophageal Adenocarcinoma. https://clinicaltrials.gov/study/NCT06043427?term=NCT06043427&rank=1.

[52] Lim H., Woo E., Hamilton T. (2018). Guideline for the Surgical Treatment of Gastric Cancer. http://www.bccancer.bc.ca/books/Documents/Gastrointestinal/BCCancer_GuidelineForTheSurgicalTreatmentOfGastricCancer.pdf.

[53] Guideline Resource Unit (2021). Gastric Cancer. Clinical Practice Guideline GI-008—Version 6. https://www.albertahealthservices.ca/assets/info/hp/cancer/if-hp-cancer-guide-gi008-gastric.pdf.

[54] Bharadwaj S., Haider K., Kakumanu S., Adnan Zaidi A., Suderman D., Rani Kanthan R., Haimanot S., Ahmed S. (2014). Provincial Esophageal Cancer and Gastro-esophageal Junction Cancer Treatment Guidelines. https://saskcancer.ca/sites/default/files/practice_guideline_document/Esophageal%20Cancer%20and%20Gastro-esophageal%20Junction%20Cancer%20Treatment%20Guidelines%20December%202018.pdf.

[55] Hebbard P., Akra M., Botkin C., Buduhan G., Cantor M., Goldenberg B. (2018). Disease Management Guideline for the Curative Treatment of Gastric Cancer. https://www.cancercare.mb.ca/export/sites/default/For-Health-Professionals/.galleries/files/treatment-guidelines-rro-files/practice-guidelines/gastro-intestinal/Guideline-for-the-curative-treatment-of-gastric-cancer-CCMB_9.pdf.

[56] Coburn N., Cosby R., Klein L., Knight G., Malthaner R., Mamazza J., Mercer D., Ringash J., Surgical Management of Gastric Cancer Guideline Development Group (2017). Staging and Surgical Approaches in Gastric Cancer. https://www.cancercareontario.ca/en/guidelines-advice/types-of-cancer/37866.

[57] Gertsen E.C., Brenkman H.J.F., van Hillegersberg R., van Sandick J.W., van Berge Henegouwen M.I., Gisbertz S.S., Luyer M.D.P., Nieuwenhuijzen G.A.P., van Lanschot J.J.B., Lagarde S.M. (2021). 18F-Fludeoxyglucose-Positron Emission Tomography/Computed Tomography and Laparoscopy for Staging of Locally Advanced Gastric Cancer: A Multicenter Prospective Dutch Cohort Study (PLASTIC). JAMA Surg..

[58] de Gouw D.J.J.M., Klarenbeek B.R., Driessen M., Bouwense S.A.W., van Workum F., Fütterer J.J., Rovers M.M., Ten Broek R.P.G., Rosman C. (2019). Detecting Pathological Complete Response in Esophageal Cancer after Neoadjuvant Therapy Based on Imaging Techniques: A Diagnostic Systematic Review and Meta-Analysis. J. Thorac. Oncol..

[59] Oh Y., Kim M.S., Lee Y.T., Lee C.M., Kim J.H., Park S. (2020). Laparoscopic total gastrectomy as a valid procedure to treat gastric cancer option both in early and advanced stage: A systematic review and meta-analysis. Eur. J. Surg. Oncol..

[60] Kodera Y., Yoshida K., Kumamaru H., Kakeji Y., Hiki N., Etoh T., Honda M., Miyata H., Yamashita Y., Seto Y. (2019). Introducing laparoscopic total gastrectomy for gastric cancer in general practice: A retrospective cohort study based on a nationwide registry database in Japan. Gastric Cancer.

[61] Bartley A.N., Washington M.K., Ventura C.B., Ismaila N., Colasacco C., Benson A.B., Carrato A., Gulley M.L., Jain D., Kakar S. (2016). HER2 Testing and Clinical Decision Making in Gastroesophageal Adenocarcinoma: Guideline From the College of American Pathologists, American Society for Clinical Pathology, and American Society of Clinical Oncology. Arch. Pathol. Lab. Med..

[62] Cheung C.C., Barnes P., Bigras G., Boerner S., Butany J., Calabrese F., Couture C., Deschenes J., El-Zimaity H., Fischer G. (2019). Fit-For-Purpose PD-L1 Biomarker Testing for Patient Selection in Immuno-Oncology: Guidelines for Clinical Laboratories from the Canadian Association of Pathologists-Association Canadienne Des Pathologistes (CAP-ACP). Appl. Immunohistochem. Mol. Morphol..

[63] Torlakovic E., Lim H.J., Adam J., Barnes P., Bigras G., Chan A.W.H., Cheung C.C., Chung J.H., Couture C., Fiset P.O. (2020). “Interchangeability” of PD-L1 immunohistochemistry assays: A meta-analysis of diagnostic accuracy. Mod. Pathol..

[64] Fernandez A.I., Robbins C.J., Gaule P., Agostini-Vulaj D., Anders R.A., Bellizzi A.M., Chen W., Chen Z.E., Gopal P., Zhao L. (2023). Multi-Institutional Study of Pathologist Reading of the Programmed Cell Death Ligand-1 Combined Positive Score Immunohistochemistry Assay for Gastric or Gastroesophageal Junction Cancer. Mod. Pathol..

[65] Nuti S., Zhang Y., Zerrouki N., Roach C., Bänfer G., Kumar G.L., Manna E., Diezko R., Kersch K., Rüschoff J. (2022). High interobserver and intraobserver reproducibility among pathologists assessing PD-L1 CPS across multiple indications. Histopathology.

[66] Robert M.E., Rüschoff J., Jasani B., Graham R.P., Badve S.S., Rodriguez-Justo M., Kodach L.L., Srivastava A., Wang H.L., Tang L.H. (2023). High Interobserver Variability among Pathologists Using Combined Positive Score to Evaluate PD-L1 Expression in Gastric, Gastroesophageal Junction, and Esophageal Adenocarcinoma. Mod. Pathol..

[67] Kalpakoff M., Hund S., Musser J., Roach C., Apostolaki A., Vilardo M., Peltz L., Watts B., LaPlaca C., Tabuena-Frolli S. (2021). Intrapatient Tumor Heterogeneity in IHC Interpretation Using PD-L1 IHC 22C3 pharmDx. Appl. Immunohistochem. Mol. Morphol..

[68] Liu D.H.W., Grabsch H.I., Gloor B., Langer R., Dislich B. (2023). Programmed death-ligand 1 (PD-L1) expression in primary gastric adenocarcinoma and matched metastases. J. Cancer Res. Clin. Oncol..

[69] Ye M., Huang D., Zhang Q., Weng W., Tan C., Qin G., Jiang W., Sheng W., Wang L. (2020). Heterogeneous programmed death-ligand 1 expression in gastric cancer: Comparison of tissue microarrays and whole sections. Cancer Cell. Int..

[70] Zhou K.I., Peterson B., Serritella A., Thomas J., Reizine N., Moya S., Tan C., Wang Y., Catenacci D.V.T. (2020). Spatial and Temporal Heterogeneity of PD-L1 Expression and Tumor Mutational Burden in Gastroesophageal Adenocarcinoma at Baseline Diagnosis and after Chemotherapy. Clin. Cancer Res..

[71] Shitara K., Lordick F., Bang Y.J., Enzinger P., Ilson D., Shah M., Van Cutsem E., Xu R.H., Aprile G., Xu J. (2023). Zolbetuximab plus mFOLFOX6 in patients with CLDN18.2-positive, HER2-negative, untreated, locally advanced unresectable or metastatic gastric or gastro-oesophageal junction adenocarcinoma (SPOTLIGHT): A multicentre, randomised, double-blind, phase 3 trial. Lancet.

[72] Wainberg Z.A., Enzinger P.C., Kang Y.K., Qin S., Yamaguchi K., Kim I.H., Saeed A., Oh S.C., Li J., Turk H.M. (2022). Bemarituzumab in patients with FGFR2b-selected gastric or gastro-oesophageal junction adenocarcinoma (FIGHT): A randomised, double-blind, placebo-controlled, phase 2 study. Lancet Oncol..

[73] Xie T., Liu Y., Zhang Z., Zhang X., Gong J., Qi C., Li J., Shen L., Peng Z. (2020). Positive Status of Epstein-Barr Virus as a Biomarker for Gastric Cancer Immunotherapy: A Prospective Observational Study. J. Immunother..

